# Prognosis of psychomotor and mental development in premature infants by early cranial ultrasound

**DOI:** 10.1186/s13052-015-0135-5

**Published:** 2015-04-09

**Authors:** Yang Duan, Fu-qiang Sun, Yue-qin Li, Sheng-shun Que, Su-yan Yang, Wen-jing Xu, Wen-hong Yu, Jun-hua Chen, Ya-jie Lu, Xin Li

**Affiliations:** Department of Neonatology, Second Hospital of Tianjin Medical University, NO.23 Pingjiang road, Hexi District, Tianjin, 300211 China

**Keywords:** Brain injury, Premature infants, Cranial ultrasonography, Infant development

## Abstract

**Background:**

It is of high incidence of brain injuries in premature infants, so it is necessary to diagnose and treat the brain injury early for neonatal clinical practice. We are aimed to investigate the relationship between early postnatal cranial ultrasonography and psychomotor and mental development in prematrue infants at the age of 12 months.

**Methods:**

Two-hundred and eight premature infants were selected and underwent follow-up from January, 2007 to November, 2012. Cranial ultrasonography was performed on them. The developmental outcomes of these premature infants at the age of 12 months were assessed by the psychomotor developmental index (PDI) scale and mental development index (MDI). The relationship between ultrasonic gray-scale value and PDI and MDI was analyzed.

**Results:**

The worse prognosis for psychomotor and mental development was associated with the gestational age, Apgar score(1 min), gender, chorioamnionitis, duration of mechanical ventilation and duration of mechanic ventilation. The differences between the prognosis of psychomotor and mental development, and peri-intraventricular hemorrhage (PIVH) and periventricular white matter damage (PWMD), were statistically significant (P < 0.05). There were also significant differences between the early postnatal ultrasonic gray-scale value and prognoses of both psychomotor development and mental development (P < 0.05). There were negative correlations between ultrasonic gray-scale and both PDI and MDI (r = −0.753, P < 0.05; r = −0.764, P < 0.05).

**Conclusions:**

The early postnatal cranial ultrasonography can assist to predict the prognosis of psychomotor and mental development for premature infants. The higher grade of PIVH and PWMD was associated with the worse prognosis of psychomotor and mental development.

## Background

The incidence of premature birth is about 12% and is rising year by year. As obstetrics technology and neonatal intensive care units have become increasingly advanced, the treatments for premature infants at early stages have developed dramatically and the survival rate of premature infants has also increased significantly [1-3]. However, The incidence of brain injuries in premature infants is high, so it is essential for neonatal clinical practice to diagnose and treat the brain injury early for premature infants [4]. There are mainly two kinds of brain injury in premature infants, including periintraventricular hemorrhage (PIVH) and periventricular white matter damage (PWMD) [5,6]. PIVH and PWMD usually cause poor outcomes in premature infants, such as cerebral palsy, mental deficiency and others. In general, ultrasound is used for the diagnostic of brain injury in premature infants, while it obtains a bad prognosis.

In this study, we proposed a noninvasive, convenient, inexpensive, ultrasound diagnostic method. Different from reported qualitatively ultrasound methods, we quantificationally assessed the brain injuries of participants by introducing quantization parameters as diagnostic criteria and classification standards. The criteria and standards will quantify the type and degree of early brain injuries in premature infants. The scales can be used to evaluate the infants’ prognostic development in movement and intelligence. The analysis is attempt to find the relationship between imaging science and a prognostic index in order to provide theoretical and practical application in early clinical prognostic evaluation of craniocerebral injury in premature infants. In this study, we have retrospectively analyzed the clinical data of 216 cases of premature infants to discuss the relationship between early postnatal cranial ultrasonographic characteristics and prognosis of nervous system development.

## Methods

### General information

After obtaining the approval of the Institutional Review Board of Second Hospital of Tianjin Medical University participants were recruited from the Department of Neonatology and provided written informed consent.

The 216 cases of premature infants from January, 2007 to November, 2012 were selected. Among them, six cases died during hospitalization, and two cases were unavailable for follow-up. Therefore, the 208 cases left were included in this study. There were 110 males and 98 females, with a gestational age ranged from 26 to 36 weeks (33.08 ± 2.34 weeks) and birth weight ranged from 800 g to 3,095 g (mean 2243.70 ± 633.45 g). There were 135 cases of vaginal delivery and 73 cases of uterine-incision delivery. The inclusion criteria are that the perinatal period of infants is less than 37 weeks, 26 to 36 weeks in this study. Criteria to exclude cases includes: infants with metabolic diseases, infants with abnormal nervous system, or infants with severe deformities of other systems.

### Methods

### Cranial ultrasonography for premature infants

The cranial ultrasonography was done at bedside with the ultrasound testing device (GE Volusion i). The premature infants were placed supine; they fell asleep or were at a resting state, during which coronal and sagittal inspections through the anterior fontanelle were performed. Both the operator's hands and ultrasonic probe were disinfected with an alcohol cotton ball to prevent cross-infection.

The parameters of ultrasound testing device were as following: small convex array sector probe with frequency of 3.5-7.5 MHz and gain adjusting of 100 dB. The inspection was finished in three days after the birth of these premature infants. The sagittal images at the posterior horns of the lateral ventricle view were obtained. The abnormal echo of the white mass of the brain was indicative of periventricular white matter injury.

The classification standards of PIVH were as following [7]: Grade I: subependymal hemorrhage; Grade II: intraventricular hemorrhage; Grade III: intraventricular hemorrhage with ventricular dilatation; Grade IV: intraventricular hemorrhage with adjacent periventricular venous hemorrhagic infarction. The classification standards of PWMD is: Grade I: the echo of periventricular white matter was enhanced transiently; Grade II: the echo of periventricular white matter was enhanced with or without focal small cysts; Grade III: the echo of periventricular white matter was enhanced significantly with or without multifocal cystic changes; Grade IV: the echo of extensive white matter were enhanced significantly with or without multifocal cysts.

### Cranial ultrasonic gray-scale value measurement for premature infants

Ultrasonic anomalous area of 1 cm^2^ of each of these premature infants was chosen to calculate the average of gray-scale value for ultrasonic anomalous areas. The calculated average value was considered as a quantitative indicator for echo strength. The extreme strong echo areas were excluded from calculation. Two experienced physicians performed the gray value measurement; and the final regional gray value was expressed as the means measurement values scored by the two physicians.

### The prognosis of psychomotor and mental development of premature infants

Psychomotor developmental index (PDI) was used to measure the psychomotor ability of participants. Mental development index (MDI) was applied to assess their abilities of eye-hand coordination, sense of sight and hearing, cognition, language and others. Twelve months after the infants were discharged from the hospital, the assessments were conducted according to the PDI scale and MDI scale. The scores of MDI and PDI ranged from 0 to 114. The score <70 is considered as disorder, 70–84 as borderline disorder and 85–114 as normal [8]. Two experienced physicians jointly scored the PDI and MDI, and experts in the field approved the scores.

### Analysis of high risk factors for brain injury

Information was recorded for these premature infants, including the gestational age, birth weight, Apgar score, gender, chorioamnionitis, duration of mechanical ventilation, chronic pulmonary diseases, etc. The intrinsic relationship between these parameters and in prognosis of psychomotor and mental development of premature infants was analyzed, retrospectively.

### Statistical analysis

Data was analyzed by an SPSS software (Version 11.0; SPSS Inc.,Chicago,IL). The measurement data was expressed as mean ± SD. Comparison among groups was performed by using a *t*-test or analysis of variance. The numeration data was analyzed by a chi square test or Kruskal-Wallis H test. Muti-factor was analyzed by ordinal Logistic regression. *P* < 0.05 was considered as statistically significant.

## Results

### The relationship between clinical characteristics and prognoses of psychomotor and mental development of premature infants

These infants were all scored according to MDI and PDI score standard after the 12 months’ follow-up. Based on the scores, they were divided into normal, borderline disorder or disorder in psychomotor development and mental development, respectively. The relevant clinical information was collected at the time of delivery. The clinical information of these infants was summarized in Table 1.

**Table 1:** **Clinical information of participants (N = 208)**

	**Prognosis of psychomotor and mental development**	
	**Normal (n = 108)**	**Abnormal (n = 100)**
Maternal data		
Unmarried	3 (2.78%)	2 (2.00%)
Less than high school education	43 (39.81%)	31 (31.00%)
Perinatal data		
Antenatal steroid therapy	63 (58.33%)	58 (58.00%)
Cesarean section delivery	43 (39.81%)	37 (37.00%)
Infant birth data		
Birth weight (mean ± SD, g)	2381.37 ± 552.30	2089.16 ± 684.02
Gestational age (mean ± SD, g)	33.85 ± 2.00	32.21 ± 2.38
Male	62 (57.40%)	51 (51.00%)
Neonatal risk factors		
Chronic lung disease	6 (5.56%)	18 (18.00%)
Sepsis	22 (20.37%)	24 (24.00%)
Necrotizing enterocolitis	11 (10%)	9 (9%)
Hospital stay, median days (range)	11.27 ± 3.47(2–82)	23.15 ± 2.47(5–79)

From the data in Tables 2 and 3, we found that the gestational age, Apgar score, sex, chorioamnionitis, duration of mechanical ventilation were all associated with the prognosis of psychomotor and mental development of premature infants. The younger the gestational age, lower the Apgar score (1 min), male, chorioamnionitis, duration of mechanical ventilation and longer the duration of mechanic ventilation, the worse of prognosis was for psychomotor and mental development.

**Table 2:** **Relationship between clinical information and psychomotor development**

**Parameters**	**β**	**SE**	**Wald** ***χ*** **2**	***P***	**95%** ***CI***
Gestational age (w)	−0.312	0.119	6.853	0.009	−0.546 ~ −0.078
Birth weight (g)	0.001	0.000	1.937	0.164	0.000 ~ 0.001
Apgar score (1 min)	−2.507	0.377	44.323	0.000	−3.246 ~ −1.769
Gender (Male)	−0.824	0.372	4.907	0.027	−1.55 ~ −0.090
Chorioamnionitis	1.846	0.468	15.596	0.000	0.930 ~ 2.763
Ventilation(day)	2.456	0.579	18.006	0.000	1.321 ~ 3.590
Chronic lung disease	−0.044	0.555	0.006	0.936	−1.132 ~ 1.044

**Table 3:** **Relationship between clinical information and mental development**

**Parameters**	**β**	**SE**	**Wald** ***χ*** **2**	***P***	**95% ** ***CI***
Gestational age (w)	−0.276	0.114	5.883	0.015	−0.500 ~ −0.053
Birth weight (g)	0.000	0.000	1.030	0.310	0.000 ~ 0.001
Apgar score (1 min)	−2.381	0.371	41.243	0.000	−3.108 ~ −1.654
Gender (Male)	−1.005	0.366	7.545	0.006	−1.722 ~ −0.288
Chorioamnionitis	1.657	0.461	12.940	0.000	0.754 ~ 2.560
Ventilation (day)	2.403	0.577	17.343	0.000	1.272 ~ 3.535
chronic lung disease	−0.299	0.551	0.294	0..588	−1.379 ~ 0.782

### The relationship between ultrasonography and prognosis of psychomotor and mental development

The relationship between ultrasonography and prognosis of psychomotor and mental development is shown in Tables 4 and 5. From these two tables, we found that the incidence of psychomotor developmental disorder was as high as 87.5% and that of mental developmental disorder was 100% for premature infants with Grade IV PIVH. The incidence of psychomotor developmental disorder was 80% and that of mental developmental disorder was 70% for premature infants with Grade IV PWMD [5]. The difference between the degree of PIVH and the prognosis of psychomotor and mental development was statistically significant (P < 0.05), so was the degree of PWMD (P < 0.05).

**Table 4:** **Relationship between ultrasonography and prognosis of psychomotor development**

	**PIVH**					**PWMD**				
	**Normal (n = 132)**	**Grade I (n = 24)**	**GradeII (n = 28)**	**Grade III (n = 16)**	**Grade IV (n = 8)**	**Normal (n = 118)**	**Grade I (n = 31)**	**GradeII(n = 30)**	**Grade III(n = 19)**	**Grade IV (n = 10)**
**Normal (n = 112)**	95 (71.97%)	12 (50%)	4 (14.29%)	1 (6.25%)	0 (0)	90	14	6	2	0
						(76.27%)	(45.16%)	(20%)	10.53	0
**Borderline disorder (n = 61)**	34 (25.76%)	11 (45.83%)	10 (35.71%)	5 (31.25)	1 (12.5%)	28	14	12	5	2
						(23.73%)	(45.16%)	(40.00%)	(26.32%)	(20%)
**Disorder (n = 35) Statistic**	3 (2.27%)	1 (4.17%)	14 (50.00%)	10 (62.5%)	7 (87.5%)	0	3	12	12	8
						0	(9.68%)	(40.00%)	(63.16%)	(80.00%)
	H = 150.722, P = 0.000					H = 177.083, P = 0.000

**Table 5:** **Relationship between ultrasonography and prognosis of mental development**

	**PIVH**					**PWMD**				
	**Normal (n = 132)**	**Grade I (n = 24)**	**GradeII (n = 28)**	**Grade III (n = 16)**	**Grade IV (n = 8)**	**Normal (n = 118)**	**Grade I (n = 31)**	**GradeII (n = 30)**	**Grade III (n = 19)**	**Grade IV (n = 10)**
**Normal (n = 112)**	90 (68.18%)	12 (50%)	6 (21.43%)	0 (0)	0 (0)	86 (72.88%)	13 (41.94%)	6 (20.00%)	3 (15.79%)	0 (0)
**Borderline disorder (n = 61)**	40 (30.30%)	10 (41.67%)	12 (42.86%)	2 (12.5%)	0 (0)	24 (20.34%)	16 (19.35%)	16 (53.33%)	5 (26.31%)	3 (30%)
**Disorder (n = 35)**	2 (1.51%)	2 (8.33%)	10 (35.71%)	14 (87.50%)	8 (100%)	8 (6.78%)	2 (6.45%)	8 (26.67%)	11 (57.89%)	7 (70%)
**Statistic**	H = 150.722, P = 0.000					H = 177.083, P = 0.000				

### The relationship between cranial ultrasonic gray-scale value and prognosis of psychomotor and mental development

The early postnatal ultrasonic gray-scale values of premature infants with differing prognoses of psychomotor and mental development were all different with statistical significance (P < 0.05) (Table 6).

**Table 6:** **Ultrasonic gray-scale values of premature infants with differing prognoses**

	**Normal**	**Borderline disorder**	**Disorder**	***F value***	***P value***
PDI	97.52 ± 3.45	108.07 ± 4.97	124.57 ± 4.33	602.030	0.000
MDI	97.72 ± 3.35	107.10 ± 6.22	124.06 ± 5.26	423.998	0.000

**Figure 1:**
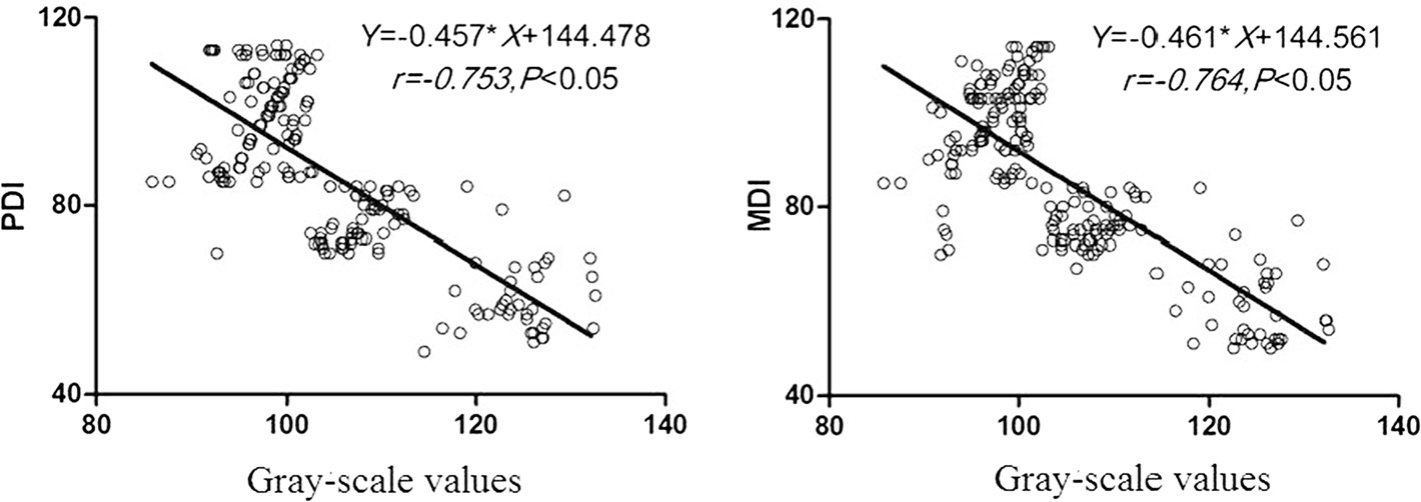
**Correlation between ultrasonic gray-scale values of premature infants and both PDI and MDI.**

According to the Pearson correlation analysis, there was a negative correlation between ultrasonic gray-scale values of premature infants and both PDI and MDI (*r* = −0.753, P < 0.05; *r* = −0.764, *P* < 0.05). Linear regression analysis was performed with PDI as the dependent variable and gray-scale value as the independent variable, and the result showed that the gray-scale value = −0.457 × PDI + 144.478 (*F* = 287.623, *P* = 0.000, *r*^2^ = 0.583, *r* = −0.753). The equation was statistically significant and has well dependencies. Another linear regression analysis was also performed with MDI as the dependent variable and gray-scale value as the independent variable. The result showed that the gray-scale value = −0.461 × PDI + 144.561 (*F* = 269.975, *P* = 0.000, *r*^2^ = 0.567, *r* = −0.764.753). It also has statistical significance and well dependencies (Figure 1).

## Discussion

As neonatal intensive therapy continues to develop, the survival rate of premature infants, especially of extremely low birth weight, is rising gradually. However, there are still many bad outcomes, such as cerebral palsy, psychomotor dysfunction and mental dysfunction [3]. There are mainly two kinds of brain injury in premature infants, including peri-intraventricular hemorrhage (PIVH) and periventricular white matter damage (PWMD). The incidence of PIVH has a close correlation with gestational age and birth weight of premature infants. A poor developmental prognosis for premature infants may often be induced by PIVH and PWMI. Thus, early, timely and accurate diagnosis is important for early intervention for premature infants. Ultrasonography is non-invasive, low-cost and is valuable in diagnosing brain injury at an early stage for premature infants [9,10].

In this study, we have analyzed retrospectively the clinical data of 216 cases of premature infants and discussed the relationship between early postnatal cranial ultrasonographic characteristics and prognosis of nervous system development.

After analyzing the relationship between clinical characteristics and prognosis of development, we found that a worse prognosis for psychomotor and mental development was associated with younger gestational age, lighter birth weight, lower Apgar (1 min) score and longer duration of mechanical ventilation. This also demonstrated that clinical characteristics of premature infants were associated with their poor prognoses. The lower Apgar score represented, the poorer conditions appeared in premature infants than in normal neonates, including muscular tension, motion, heart rate, etc. The variations of incidence of chorioamnionitis in different developmental prognoses were found to be without statistical significance. The chorioamnionitis may usually cause an intrauterine blood circulation disorder leading to intrauterine fetal anoxia. This result may be biased because the limit cases of chorioamnionitis in this study.

A research found a correlation between acute pulmonary injury and brain white matter injury. The assisted ventilation treatment for respiratory system diseases may be an important cause of brain white matter injury in the premature infant [8]. In the study of Saliba et al., they demonstrated that periventricular leucomalacia (PVL) and cerebral palsy were associated with long term and excessive ventilation, which may induce hypocapnia, leading to vasoconstriction, and then injure the white matter [11]. These are consistent with our findings. According to the report, the incidence of PIVH was about 20%-40% in premature infants with birth weight lower than 1500 g and gestational age younger than 32 weeks [12]. Limperopoulos et al. also found that almost all of the premature infants with birth weight lower than 700 g had PIVH of different degrees [13]. Periventricular whiter matter injury is also a common brain injury of premature infants. Its typical manifestation is PVL. As with PIVH, PVL also tends to occur in premature infants with a gestational age of 22–25 weeks. The younger the gestational age was, the higher of incidence of brain injury was. Researchers also demonstrated that the PVL incidence was higher in premature infants who underwent assisted ventilation than in those who didn't, and that in the extremely low birth weight premature infants, with an incidence of about 4-7% [14].

The occurrence of PIVH is associated with germinal matrix. It’s located beneath the ependymal layer, mainly consisting of primitive cells from the junction region of the lateral ventricle near the head of the caudate nucleus and thalamus. When the gestational age is 26–32 weeks, the germinal matrix is most obvious [15]. In this stage, the germinal matrix has a loose structure, thin vascular wall and increased cerebral venous pressure. Hypoxia and hypercapnia may induce bleeding of the germinal matrix beneath the ependymal layer. The blood can enter the ventricle via the ruptured ependymal layer and cause ventricular hemorrhage [16-18]. In the premature infant, the cerebral vessels are immature, the terminal arteries distribute in the periventricular region, and the vascular self-regulation system is vulnerable; thus, such infants are susceptible to ischemic damage or reperfusion, leading to PWMD. The regional cerebral blood flow and metabolic demand have close relationships with the cerebral vascular structures and functional developments. They are the important pathogenic factors for hypoxic ischemic brain damage [19-21].

In this study, we also found that the premature infants who suffered from more severe PIVH and PWMD by early postnatal cranial ultrasonography, had a poorer prognosis of psychomotor and mental development. In premature infants with Grade IV PIVH, the incidence of psychomotor developmental disorder was 87.5% and that of mental developmental disorder was 100%. In premature infants with Grade IV PIVH, the incidence of psychomotor developmental disorder was as high as 87.5% and that of mental developmental disorder was 70%. We have also calculated the ultrasonographic average gray-scale values for premature infants and analyzed their relationship with the prognosis of MDI and PDI. We found the negative correlation between ultrasonic gray-scale values and PDI and MDI. This reveals that the higher gray-scale value is associated with the poorer prognosis of psychomotor and mental development.

The brain development is initiated from the ectoderm, then the neurons proliferate at the third to fourth months and migrate at the fourth to fifth months, and the nerve cell formation occurs after the fifth month, while the white matter myelination is completed after birth [22,23]. The brain development runs throughout the whole process of fetal development, especially the later stage of fetal development. As the developmental speed and degree are different in neuron arrangement, orientation and stratification, as well as formation of synapses, the ultrasonic gray-scale values of them vary. Therefore, the gray-scale value of echo can reflect the damage degree of the injured region. This has provided a theoretical support for our study. Since a limited number of cases will be selected in this study, some bias is expected in the results. We plan to expand the number of cases in future studies to help reduce all kinds of bias and to improve the reliability of the results. Moreover, we only carry out analysis on early phase ultrasound results and not mid-late phase. The mid-late phase ultrasound detection will be used in our future research.

In conclusion, brain injury commonly occurs in premature infants, and the PIVH and PWMD of premature infants are associated with the prognosis of psychomotor and mental development. The early postnatal cranial ultrasonography is a non-invasive and accurate method for determination of the brain injury degree for premature infants. Therefore, it is valuable in predicting the prognosis of development for premature infants.

## Abbreviations

MDI: Mental development index
PDI: Psychomotor developmental index
PIVH: Peri-intraventricular hemorrhage
PWMD: Periventricular white matter damage
PVL: Periventricular leucomalacia
